# Association of Traumatic Dental Injuries with Individual-, Sociodemographic- and School-Related Factors among Schoolchildren in Midwest Brazil

**DOI:** 10.3390/ijerph110909885

**Published:** 2014-09-22

**Authors:** Maria do Carmo Matias Freire, Daniela Nobre Vasconcelos, Alessandra dos Santos Vieira, Júlia Arantes Araújo, Rafael da Silveira Moreira, Maria de Fátima Nunes

**Affiliations:** 1 Department of Stomatological Sciences, Faculty of Dentistry, Federal University of Goias, Primeira Avenida, Setor Universitario, Goiania, GO 74605-220, Brazil; E-Mails: danynv@hotmail.com (D.N.V.); alessandra_dsv@hotmail.com (A.S.V.); julia_odonto@hotmail.com (J.A.A.); nunes.mariadefatima@gmail.com (M.F.N.); 2 Department of Public Health. Aggeu Magalhães Research Center. Oswaldo Cruz Foundation, Ministry of Health, Av. Professor Moraes Rego, s/n, Campus da UFPE, Cidade Universitária, Recife, PE 50670-420, Brazil; E-Mail: saudepopular@yahoo.com.br

**Keywords:** dental trauma, sociodemographic factors, schoolchildren

## Abstract

The objective of this study was to assess the association of untreated traumatic dental injuries (TDI) with individual-, sociodemographic- and school-related factors among 12-year-old schoolchildren in Midwest Brazil. This cross-sectional study was carried out in 2010 in the city of Goiania, Brazil. A random sample of 2075 schoolchildren was examined and interviewed. Untreated TDI in the permanent incisors was assessed using the methodology of the Brazilian National Oral Health Survey. Rao-Scott test and multinomial logistic regression were used to analyze the associations between independent variables and three categories of TDI, using a hierarchical method. Independent variables were children’s sex, self rated color/race and size of incisal overjet, their mother’s level of schooling, and the schools’ type and geographic location. The prevalence of trauma was 17.3% (CI 95% = 15.2–19.4); enamel fractures were the most common TDI (13.1%). In the adjusted model, a higher chance of having two or more teeth with TDI was found among boys, those whose mothers had lowest level of schooling, and those attending schools located in health districts with lower socioeconomic indicators. It was concluded that the prevalence of TDI was low and that it was associated with individual factors as well as the school environments.

## 1. Introduction

Traumatic dental injuries (TDI) occur mainly in childhood and adolescence. They have impact on individuals, families and society, as TDI have psychological effects and impacts on quality of life [1,2,3]. National and international studies on the etiology of dental trauma in the permanent dentition indicate that the most frequent type of injury is a simple crown fracture of the maxillary central incisors. Oral factors, environmental determinants and human behaviour increase the risk of TDI [4]. Males experience more dental trauma than females, and accidents at home and school are the major sources of TDI [5]. Inadequate lip coverage and incisal overjet greater than 5 mm are associated with the occurrence of dental injury [4].

Although there is no consensus on TDI as a major public health problem, the increasing levels of traffic accidents and other types of violence as well as participation of children and adolescents in high risk physical activities may lead to increasing levels of TDI [6] and its complex and costly outcomes for the public health services. Therefore, studies aiming to investigate the social determinants of trauma are needed to inform health promotion strategies to prevent its occurrence.

Despite the growing scientific interest on the epidemiology of TDI, knowledge about its socioeconomic determinants is still scarce. Reviews on the subject showed that there were few studies correlating traumatic injuries in permanent teeth and socioeconomic indicators and the majority found inconsistent associations [7,8]. That may be related to the indicators used. Results also vary between countries, since lifestyles and levels of economic development may influence the occurrence of trauma in the population.

Several studies on TDI have been carried out among schoolchildren. Most of them have focused on individual demographic and clinical variables rather than on factors related to the school environment and other social determinants. Few studies have tried to assess the influence of the type of school as an indicator of socioeconomic status. This is particularly important in developing countries, where children from high socioeconomic backgrounds usually attend private schools and those from low socioeconomic backgrounds, public schools. Results of studies comparing rates of TDI and type of schools have been inconsistent [9,10,11,12,13,14]. Furthermore, the influence of the schools’ geographic location is seldom investigated [15,16]. We have hypothesized that the school environments as measured by their geographic location are influential socioeconomic factors to the occurrence of dental trauma, which may interact with individual factors. The objective of this study is to assess the association of untreated traumatic dental injuries with individual-, sociodemographic- and school-related factors among 12-year-old schoolchildren in Midwest Brazil.

## 2. Methods

This cross-sectional study used data from a broader oral health survey, carried out in schools of the urban area in the city of Goiania (Midwest Brazil) in 2010. The research protocol was approved by the Ethics Committee of the Federal University of Goias, Brazil (Protocol 226/2010), based on the Resolution 196/96 from the National Health Board.

### 2.1. Participants

Sample size was calculated to be representative of the 12-year-old schoolchildren in Goiania. We used cluster sampling technique in two stages: schools and schoolchildren. Because many conditions were investigated in the broader-scoped population survey, sample size calculation was based on the outcome with the highest required sample size (dental caries). It was calculated using an equation for proportions for infinite populations based on caries prevalence, using the Epi Info^©^ software, version 3.5.1 (Centers for Disease Control and Prevention, Atlanta, GA, USA). The minimum number of schoolchildren that needed to participate was 2171, considering a confidence interval of 95%, sampling error of 2%, and caries prevalence of 65.3%. For effect of study sample design, a simplified and conservative correction was needed, multiplying the obtained sample size by 1.2 (an extra 20%). The final sample size was 2605 schoolchildren. To calculate the number of schools (*N* = 41), a formula was used that multiplied the number of schools by the number of schoolchildren of the sample, divided by the total number of 12-year-old schoolchildren in Goiania. Both schools and children were drawn from lists provided by the local education authorities.

### 2.2. Data Collection

Individual data were collected by six previously trained and calibrated dentists. Inter-examiner Kappa coefficient for dental trauma varied from 0.92 to 1.0, showing an almost perfect agreement. Oral health examinations were carried out at the schools using a mouth mirror and a WHO periodontal probe under natural light, with the children seated in school chairs. Presence of clinical evidence of untreated dental injuries in the crowns of upper and lower permanent incisors was assessed according to the methodology of the 2010 Brazilian National Oral Health Survey [17]. The following conditions were registered: fracture involving the enamel only, fracture involving enamel and dentine, fracture involving enamel and dentine with pulp exposure, and missing tooth due to trauma. Missing teeth were the result of avulsion or other TDI which necessitated extraction.

Overjet size was measured with incisors in the occlusal position and the probe positioned parallel to the occlusal plane. The distance, in millimeters, between the buccal surface of the more prominent upper central incisor and the corresponding lower incisor was registered [17].

The following information on sociodemographic characteristics of the participating children was recorded: sex, self-rated skin color or race, and mother’s level of schooling. Self-rated skin color or race used criteria proposed by the Brazilian Institute of Geography and Statistics [18]. Mother’s level of schooling was obtained from the children´s school records, and was based on completed years of study. Data regarding the schools were obtained from the local Education and Health Authorities: type of school (public and private) and the city’s Health Districts where the schools were located.

### 2.3. Statistical Analysis

**Figure 1:**
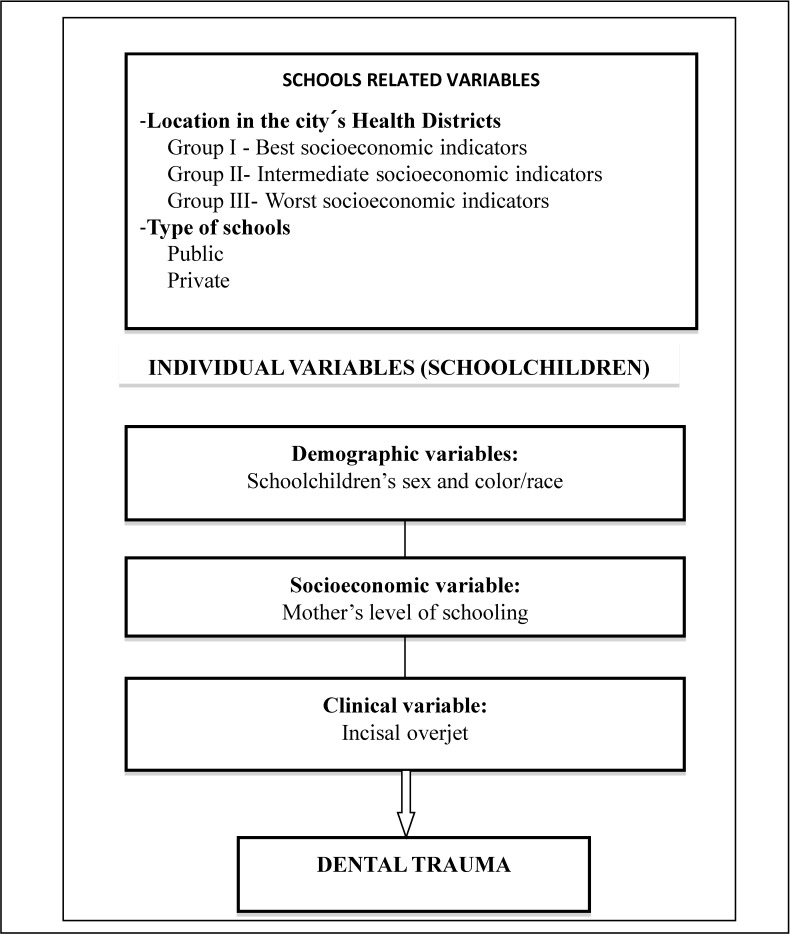
Theoretical hierarchical proposed model of the association between individual and schools related factors and dental trauma of 12-year-old schoolchildren.

Statistical analysis used a hierarchical approach (Figure 1), as proposed by Victora *et al.* [19]. The dependent variable was the prevalence of dental trauma (TDI), which was stratified in three categories according to the number of teeth involved: no trauma; trauma in one tooth only; and trauma in two or more teeth.

Independent variables were divided into two hierarchical blocks of data organization from distal to proximal determinants of TDI: variables related to the schools and those related to the schoolchildren. In the individual block, we analyzed children’s demographic characteristics, one socioeconomic indicator (mothers’ level of schooling) and one clinical variable (incisal overjet).

In the second block, variables related to the schools were type (public and private) and city’s Health District where schools were located. The seven districts were grouped according to their socioeconomic characteristics, as informed by the local Health Authority: Group I; the best indicators (Central); Group II; intermediate indicators (North, South and East); and Group III; the worst socioeconomic indicators (Southwest, West and Northwest).

Descriptive statistics was initially performed for the outcome variable. Then all independent variables were described according to the three categories of dental injuries (no trauma; trauma in one tooth only; and trauma in two or more teeth). Rao-Scott test, an equivalent to Chi-square test for complex samples, was used to test dependence between variables. This analysis was performed using the Stata 12^©^ software. Multinomial logistic regression was then used to test the associations between the independent variables and the three categories of traumatic dental injury. This is a useful technique to cases where the outcome can have three or more possible types, making it possible to predict probabilities of multiple outcome categories simultaneously, compared with a common reference category. Odds ratios and 95% Confidence Interval (CI) were calculated, and comparisons were made between each independent variable and each injury category, using the non-occurrence of trauma as the reference category. The variables that showed association with TDI were included in the multiple multinomial logistic regression model, adjusting the individual variables for those related to the schools. For all analysis, the 5% significance level and sample weights derived from the complex sample design (school weights) was used, using the command *svy* in Stata.

## 3. Results

Of the 41 schools invited to participate, 39 (24 public and 15 private) accepted. Of the 2962 schoolchildren invited to take part, 2075 agreed and were examined (response rate = 70.0%). The main reasons for non-response were lack of permission from the parents and children’s absence from schools during the data collection period.

Sample was composed mainly of males (50.9%), children who classified themselves as brown (54.5%), and those whose mothers had studied from eight to eleven years (51.2%). Most of the students were from public schools (71.2%). The overall prevalence of untreated TDI was 17.3% (CI = 15.2–19.4). The most common type of injury was fracture involving the enamel only (13.1%; CI = 11.4–15.2), followed by fracture involving enamel and dentine (3.8%; CI = 2.9–4.9). The other conditions were rare: fracture with pulp exposure (1.0; CI = 0.6–1.7) and missing tooth due to trauma (0.1%; CI = 0.0–0.4). The maximum number of damaged teeth per individual was four, and varied according to the type of injury. Most of the children who had experienced traumatic dental injuries (*N* = 358) had only one tooth with TDI (*N* = 254; 12.2%), 90 (4.3%) had two, 10 (0.5%) had three and only 4 (0.2%) had four teeth with TDI.

Results of bivariate analyses showed significant differences in the distribution of the outcome (number of teeth affected) by the schoolchildren’s sex and the two factors related to the schools (type and location) (Table 1). Trauma in at least one tooth was more frequent among boys (*p* < 0.01), those attending public schools compared to private ones, and schools in Health Districts II and III compared to Health District I (*p* < 0.05).

**Table 1:** Frequency distribution of untreated dental trauma according to independent variables.

Individual Variables				Dental Trauma	
		N (%) ^a^	No trauma % (95% CI) ^a^	Trauma in one tooth % (95% CI) ^a^	Trauma in two or more teeth % (95% CI) ^a^
Sex *p* = 0.0005 ^b^	Female	1053 (50.9)	85.4 (83.1–87.4)	11.1 (9.4–12.9)	3.5 (2.6–4.7)
	Male	1022 (49.1)	80.6 (77.7–83.1)	13.4 (11.7–15.3)	6.0 (4.6–7.8)
Color/race *p* = 0.6781	White	787 (36.4)	82.9 (79.6–85.8)	12.6 (10.3–15.3)	4.5 (3.1–6.4)
	Black	192 (8.8)	82.0 (78.1–85.4)	14.3 (11.1–18.3)	3.7 (2.0–6.4)
	Brown	1089 (54.5)	83.1 (79.5–86.1)	11.7 (9.8–13.9)	5.2 (3.6–7.4)
Mother’s level of schooling (years)	More than 11	427 (21.3)	83.4 (80.2–86.2)	13.4 (10.9–16.4)	3.2 (1.8–5.3)
*p* = 0.0725	8 to 11	1080 (51.2)	83.3 (80.3–85.9)	12.1 (10.1–14.5)	4.6 (3.4–6.1)
	Less than 8	568 (27.5)	82.0 (79.1–84.5)	11.5 (9.8–13.5)	6.5 (5.0–8.5)
Size of incisal overjet	5 mm or less	1910 (91.7)	83.4 (81.2–85.3)	12.0 (10.7–13.5)	4.6 (3.7–5.8)
*p* = 0.2787	6 mm or more	165 (8.3)	78.6 (70.1–85.3)	14.5 (8.8–22.8)	6.9 (3.9–11.9)
**Variables related to the schools**					
Health District (Group)	I	425 (18.4)	86.8 (84.6–88.8)	11.3 (8.7–14.5)	1.9 (1.1–3.3)
*p* = 0.0469 ^b^	II	745 (37.9)	81.8 (77.2–85.6)	13.1 (10.5–16.3)	5.1 (3.7–7.0)
	III	905 (43.7)	82.3 (79.4–85.0)	11.9 (10.2–13.8)	5.8 (4.2–7.7)
Type of school *p* = 0.0192 ^b^	Private	604 (28.8)	85.8 (82.4–88.7)	11.5 (8.9–14.7)	2.7 (1.6–4.3)
	Public	1471 (71.2)	81.8 (79.1–84.3)	12.5 (11.0–14.3)	5.7 (4.4–7.1)
Total			83.0 (80.7–85.0)	12.2 (10.9–13.7)	4.8 (3.8–6.0)

^a^ Unweighted count for N. Sample weight was considered for % and 95% CI; ^b^ Rao-Scott test. *p* < 0.05.

The results of non-adjusted multinomial logistic regression analysis showed significant associations between untreated dental injuries and the two variables regarding the school context, and also between injuries and the schoolchildren’s sex and maternal schooling (Table 2).

**Table 2:** Non-adjusted multinomial logistic regression analysis for the association between untreated dental trauma and individual and school related factors.

Variables Related to the Schools		Dental Trauma ^a^			
		Trauma in one tooth OR (95% CI)	*p*-value	Trauma in two or more teeth OR (95% CI)	*p*-value
Health District (Group)	I	1.00	---	1.00	---
	II	1.23 (0.84–1.81)	0.28	3.14 (1.63–.06)	<0.01
	III	1.11 (0.80–1.54)	0.52	3.22 (1.71–6.06)	<0.01
Type of school	Private	1.00	---	1.00	---
	Public	1.14 (0.82–1.58)	0.41	2.20 (1.11–3.61)	<0.01
Individual variables					
Sex	Female	1.00	---	1.00	---
	Male	1.28 (1.05–1.56)	0.02	1.76 (1.24–2.49)	<0.01
Color/race	White	1.00	---	1.00	---
	Black	1.15 (0.76–1.73)	0.50	1.03 (0.57–1.86)	0.92
	Brown	0.93 (0.67–1.27)	0.64	1.15 (0.66–2.04)	0.61
Mother’s level ofschooling (years)	More than 11	1.00	---	1.00	---
	8 to 11	0.90 (0.67–1.20)	0.48	1.44 (0.77–2.66)	0.24
	Less than 8	0.87 (0.64–1.17)	0.37	1.97 (1.09–3.57)	0.02
Size of incisal overjet	5 mm or less	1.00	---	1.00	---
	6 mm or more	1.27 (0.72–2.23)	0.39	1.53 (0.85–2.74)	0.15

^a^ No trauma as reference category; OR (95% CI) = Odds Ratio and 95% CI.

Boys had a higher chance of having a TDI in one (OR = 1.28; CI = 1.05–1.56) or in two or more teeth (OR = 1.76; CI = 1.24–2.49), compared to the non-occurrence of a TDI. Those whose mothers had the lowest level of schooling (OR = 1.97; CI = 1.09–3.57), who attended public schools (OR = 2.20; CI = 1.11–3.61), and those located in Health Districts II (OR = 3.14; CI = 1.63–6.06) and III (3.22; CI = 1.71–6.06) had a higher chance of having a TDI in two or more teeth. Multiple multinomial logistic regression analysis was performed, including all variables that showed association with TDI in the non-adjusted analysis and also the variable type of school (Table 3).

**Table 3:** Adjusted multinomial logistic regression analysis for the association between dental trauma and individual and school related factors.

Variables Related to the Schools		Dentral Trauma ^a^			
		Trauma in one tooth OR (95% CI)	*P*-value	Trauma in two or more teeth OR (95% CI)	*P*-value
Health District (Group)	I	1.00	---	1.00	---
	II	1.17 (0.80–1.70)	0.40	2.69 (1.40–5.16)	<0.01
	III	1.01 (0.68–1.52)	0.94	2.46 (1.24–4.85)	0.01
Type of school	Private	1.00	---	1.00	---
	Public	1.15 (0.77–1.73)	0.47	1.57 (0.85–2.89)	0.14
Individual variables ^b^					
Sex	Female	1.00	---	1.00	---
	Male	1.28 (1.05–1.56)	0.02	1.75 (1.25–2.47)	<0.01
Mother’s level of schooling (years)	More than 11	1.00	---	1.00	---
	8 to 11	0.92 (0.69–1.21)	0.53	1.48 (0.79–2.76)	0.21
	Less than 8	0.87 (0.65–1.17)	0.37	1.97 (1.08–3.60)	0.03

^a^ No trauma as reference category; ^b^ Adjusted for the contextual variables; OR (95% CI)= Odds Ratio and 95% CI.

Results showed that TDI remained associated with the schoolchildren’s sex and their mothers’ education after controlling for the school variables. In the adjusted model, those attending schools located in Health Districts II (with intermediate socioeconomic indicators) and III (with the worst indicators), compared to Health District I (with the best indicators) were 2.69 (CI = 1.40–5.16) and 2.49 (CI = 1.24–4.85) respectively more likely to have a TDI in two or more teeth. Boys were also more likely to have one tooth with TDI (OR = 1.28; CI = 1.05–1.56) or two or more teeth with that condition (OR = 1.75; CI = 1.25–2.47), compared to girls. Those whose mothers had less than eight years of schooling, compared to the highest level also had more chance of having a TDI in two or more teeth (OR = 1.97; CI = 1.08–3.60).

## 4. Discussion

The occurrence of untreated TDI was influenced by the interaction between individual and school environments, confirming our hypothesis. The findings showed that TDI was more frequent among boys and those from lower socioeconomic backgrounds, as measured by their mothers’ level of schooling. The school environment influenced occurrence of TDI, since a higher number of affected teeth was found in schools in health districts with the worst socioeconomic characteristics.

Results on differences between males and females are in accordance with previous studies [5,13,14,16]. Regarding the influence of socioeconomic status, although there is evidence that accidents are more common among less privileged groups [20], there is no consensus in the literature that they experience more dental trauma [7,8]. A previous study among Brazilian schoolchildren showed that those with mothers with higher levels of schooling had more TDI than those with less well educated mothers [21], but others have not found such association [13,14,22,23,24]. Results on the relationship between dental trauma and other indicators of the family socioeconomic status are also inconsistent [14,21,22,23,25,26,27,28,29].

Possible explanations for the links between trauma and sociodemographic factors include that the risky behaviours are more common among boys and those children from more deprived areas [4]. There is also evidence that adolescents who had adverse family environments along the life course had more traumatic dental injuries than those who experienced more favorable family environments [30].

Our findings on the relationship between trauma and the schools location corroborates previous research in the same Brazilian region, showing lower prevalence of injuries in school neighborhoods with higher levels of social capital [15]. Similar findings were reported in a city in Southern Brazil, where the schoolchildren’s place of residence was analyzed and a higher prevalence was found in poorer areas [16,31]. The present study has also shown that the type of school is not related to TDI after controlling for the other variables, as shown in other studies [12,14,16]. However, there is no consensus regarding this relationship, since some studies have reported that injuries are more common in private schools [9,10] and by other, in public schools [11]. Considering that accidents at the schools are one of the main location for TDI, future studies should include other characteristics of the school environment that would help to clarify the possible differences between public and private institutions, since in Brazil they are a proxy of the families’ socioeconomic levels. Furthermore, private schools, where children from upper social class usually study, are generally located in the more privileged health districts. This may explain why the adjusted results showed no association between type of school and dental trauma, since both variables (type and location) are related to socioeconomic status.

The prevalence of dental trauma in Goiânia (17.3%) was lower than the overall prevalence in Brazil (20.5%) in the same age group and using the same methodology [17]. It was also lower than in some Brazilian cities [11,13,14,21,22,23,24] and higher than in others [22,26]. It is important to mention that, in the present study, treated TDI were not included, and this may have underestimated the prevalence. The main type of TDI was fracture involving enamel only, as reported in most previous studies worldwide [5]. Such comparisons should be made with caution, since the studies have used different methodologies.

Our findings on the possible influence of incisal overjet differ from most previous studies, which showed that an overjet higher than 5 mm increases the risk of TDI [4]. Findings were, however, similar to that reported in other Brazilian cities [13,21,22].

This was a population based study in a representative sample of 12-year-old schoolchildren, with a good response rate. Because it was carried out in a local population, the findings cannot be generalized to other groups. One important contribution was that it has confirmed the importance of the schools location as indicators of inequalities in dental trauma distribution within urban areas. Also, the outcome variable provided detailed information on prevalence of TDI, since it has considered the number of teeth affected and not only the total trauma experience. Among the limitations is the lack of information regarding the causes and other aspects that may influence the occurrence of TDI, as well as data on treated teeth and treatment needs. Furthermore, a causal relationship cannot be established, since this is a cross-sectional study.

Since the less privileged groups have a higher risk of TDI and accidents in the school playground frequently occur at school, this may have implications for the public health system as well as the education authorities. There is evidence that health promoting schools, which have a more comprehensive curriculum and commitment towards health and safety, have significantly lower levels of TDI [32]. Therefore, efforts should be made to reduce the occurrence of injuries that are amenable to preventive measures, and adequate treatment should be provided to those in need, in order to minimize dental trauma lifelong consequences.

## 5. Conclusions

It was concluded that most of the injuries among the 12-year-old schoolchildren in Goiânia were of small magnitude, and trauma prevalence was associated with individual factors as well as the school environment.
